# Laminar Distribution of Subsets of GABAergic Axon Terminals in Human Prefrontal Cortex

**DOI:** 10.3389/fnana.2018.00009

**Published:** 2018-02-16

**Authors:** Kenneth N. Fish, Brad R. Rocco, David A. Lewis

**Affiliations:** ^1^Western Psychiatric Institute and Clinic, Department of Psychiatry, University of Pittsburgh School of Medicine, Pittsburgh, PA, United States; ^2^Department of Neuroscience, University of Pittsburgh School of Medicine, Pittsburgh, PA, United States

**Keywords:** GAD65, GAD67, glutamic acid decarboxylase, vGAT, human PFC

## Abstract

In human prefrontal cortex (PFC), ~85% of γ-aminobutyric acid (GABA)-expressing neurons can be subdivided into non-overlapping groups by the presence of calbindin (CB), calretinin (CR) or parvalbumin (PV). Substantial research has focused on the differences in the laminar locations of the cells bodies of these neurons, with limited attention to the distribution of their axon terminals, their sites of action. We previously reported that in non-human primates subtypes of these cells are distinguishable by differences in terminal protein levels of the GABA synthesizing enzymes glutamic acid decarboxylase 65 (GAD65) and GAD67. Here we used multi-label fluorescence microscopy in human PFC to assess: (1) the laminar distributions of axon terminals containing CB, CR, or PV; and (2) the relative protein levels of GAD65, GAD67 and vesicular GABA transporter (vGAT) in CB, CR and PV terminals. The densities of the different CB, CR and PV terminal subpopulations differed across layers of the PFC. PV terminals comprised two subsets based on the presence of only GAD67 (GAD67+) or both GADs (GAD65/GAD67+), whereas CB and CR terminals comprised three subsets (GAD65+, GAD67+, or GAD65/GAD67+). The densities of the different CB, CR and PV GAD terminal subpopulations also differed across layers. Finally, within each of the three calcium-binding protein subpopulations intra-terminal protein levels of GAD and vGAT differed by GAD subpopulation. These findings are discussed in the context of the laminar distributions of CB, CR and PV cell bodies and the synaptic targets of their axons.

## Introduction

The questions “Why does the cortex have layers? What is the function of layering? How do cortical neurons integrate information across different layers?” are particularly challenging to answer for the human neocortex because it is not possible to perform the types of tract tracing and electrophysiological studies that have provided key insights in other species. However, important information about human cortical neurons and their axon terminals can be obtained using techniques, such as quantitative fluorescence microscopy, that can be employed in postmortem human brain.

The release of γ-aminobutyric acid (GABA) from the axon terminals of interneurons plays a critical role in regulating the activity of excitatory pyramidal cells (PCs) and thus in determining the function of cortical networks. Essentially all cortical GABA is synthesized locally in terminals by the 67 and 65 kilodalton isoforms of glutamic acid decarboxylase (GAD), which are products of separate genes and undergo different post-translational modifications. GAD65 is thought to provide the on-demand pool of GABA, whereas GAD67 provides the basal pool. GAD65 has a long half-life (>24 h) and is efficiently trafficked to axonal terminals; in contrast, the half-life of GAD67 is short (~2 h) and its trafficking to terminals appears to be less efficient as it is distributed throughout interneurons. GAD activity is regulated by a cycle of activation and inactivation, which is determined by the binding and release, respectively, of its co-factor, pyridoxal 5′-phosphate. The activity of GAD65 is co-factor dependent and is highly regulated in response to GABA concentration and neuronal activity, and thus under steady state conditions GAD65 is largely inactive (~70 to 93%). In contrast, GAD67 is primarily active (~72%) and is activity-regulated mainly by transcription. In concert, these findings suggest that the two GAD isoforms: (1) provide complementary means for regulating GABA synthesis; and (2) are involved differentially in the spatial and temporal processing of information by GABA-containing neurons (Wilson and Groves, 1981; Contreras et al., 1992; Mercugliano et al., 1992; Feldblum et al., 1993, 1995; Esclapez et al., 1994; Pedneault and Soghomonian, 1994; Soghomonian et al., 1994; Wilson and Kawaguchi, 1996; Bowers et al., 1998; Soghomonian and Martin, 1998).

Based on their functional properties and synaptic targets, different classes of cortical GABAergic neurons play complementary roles in regulating the output of PCs in each cortical layer. Most (~85%) GABAergic neurons in the primate prefrontal cortex (PFC) can be differentiated into non-overlapping subtypes based on the expression of one of three calcium-binding proteins—parvalbumin (PV; ~20%), calbindin (CB; ~20%), or calretinin (CR; ~45%; Condé et al., 1994; del Río and DeFelipe, 1996; Gabbott and Bacon, 1996; Barinka and Druga, 2010). Terminals from PV neurons target the perisomatic region of PCs and are thought to play an important role in generating gamma oscillations (Gonzalez-Burgos et al., 2015). Terminals from a subset of CB neurons target dendritic compartments of PCs and of other GABAergic, non-CB neuron subtypes (Lewis et al., 2002). Thus, PV and CB neurons provide different strategies for regulating neuronal input-output transformations within cortical circuits as well as feedforward or feedback inhibition within and between cortical layers. Terminals from CR neurons mainly target GABAergic neurons and mediate disinhibitory control of PCs, leading to the selective amplification of local signal processing (Melchitzky and Lewis, 2008). Interestingly, PV, CB and CR interneuron subtypes in monkey PFC can be further subdivided based on the expression of GAD65—some express both GAD65 and GAD67, whereas others express only GAD67 (Fish et al., 2011; Rocco et al., 2016b). Considering the unique role GAD65 and GAD67 play in GABA synthesis within terminals, differential expression of GAD might define functional subsets of PV, CB and CR neurons.

To gain insights about human cortical neurons and their axon terminals in the context of the lamination of the human PFC, the present study capitalized on new quantitative fluorescence microscopy techniques to assess indicators of the amount and type of GABA inhibition each cortical layer receives. Because GAD65 and GAD67 play unique roles in the synthesis of terminal GABA, a major focus of the studies was the assessment of GAD protein levels in terminals immunoreactive (IR) for CB, CR, or PV within each layer.

## Materials and Methods

### Subjects

Brain specimens from 20 subjects (Supplementary Table S1) were recovered during autopsies conducted at the Allegheny County Medical Examiner’s Office (Pittsburgh, PA, USA) after obtaining consent from the next of kin. An independent committee of experienced research clinicians confirmed the absence of any psychiatric or neurological diagnoses for each subject using the results of structured interviews conducted with family members, review of medical records and neuropathology exam. Because the length of the postmortem interval (PMI) can affect protein integrity and aging can differentially affect gene expression, only subjects with PMI <16 h and age ≤55 years were used. The University of Pittsburgh’s Committee for the Oversight of Research and Clinical Training Involving the Dead and Institutional Review Board for Biomedical Research approved all procedures.

The left hemisphere of each brain was blocked coronally at 1–2 cm intervals, immersed in 4% paraformaldehyde for 48 h at 4°C and then washed in a series of graded sucrose solutions and cryoprotected. Tissue blocks containing the PFC were sectioned coronally at 40 μm on a cryostat and stored in a 30% glycerol/30% ethylene glycol solution at −30°C until processed for immunohistochemistry.

### Immunohistochemistry

Four different quadruple-label immunohistochemistry experiments were performed (Table 1). For each subject, two sections containing PFC area 9, identified from nearby Nissl-stained sections, and spaced ~500 μm apart were used in each of the four experiments. A single run containing 40 sections (2 sections/subject) was performed for each experiment. The sodium citrate antigen retrieval method (Jiao et al., 1999) was performed to enhance immunostaining followed by section permeabilization with 0.3% Triton X-100 in PBS for 30 min at room temperature (RT). Sections were then blocked using 20% donkey serum in PBS for 2 h at RT, and incubated for ~72 h at 4°C in PBS containing 2% donkey serum and primary antibodies (Table 1). The specificity of each antibody was verified as follows: (1) Western blot using human PFC tissue; (2) PV, CB, CR and vesicular GABA transporter (vGAT) antibodies were analyzed by immunolabeling and Western blot using control mouse and knockout tissue; (3) PV (rabbit), CB and CR antibodies were verified using immunolabeling after preadsorption with recombinant protein; and (4) PV (guinea pig), vGAT and GAD antibodies were verified by immunolabeling after preadsorption with the peptide against which each were raised (data not shown, manufacture data sheets, and Celio and Heizmann, 1981; Gottlieb et al., 1986; Kagi et al., 1987; Chang and Gottlieb, 1988; Schwaller et al., 1993; Airaksinen et al., 1997; Guo et al., 2009). Sections were then rinsed for 2 h in PBS and incubated for 24 h in PBS containing 2% donkey serum and secondary antibodies (donkey host) conjugated to biotin (1:250), Alexa 488 (1:500), Alexa 568 (1:500), or Alexa 647 (1:500; Invitrogen, Grand Island, NY, USA for all Alexa secondary antibodies) at 4°C. Next, the sections were rinsed in PBS (2 h), incubated with streptavidin Alexa 405 (1:200) for 24 h, rinsed in PBS (2 h) and mounted (ProLong Gold antifade reagent, Invitrogen) on slides which were stored at 4°C until imaged. Secondary antibody specificity was verified by omitting the primary antibody in control experiments. Multiple pilot studies were performed to determine if any primary/secondary combinations influenced the outcome; results from these studies indicated that the ability to detect each antigen was not dependent on the secondary antibody spectra.

**Table 1:** Antibodies and immunohistochemistry experiments.

				Experiments			
Antigen	Species	Dilution	Source	A	B	C	D
CB	Rabbit	1:1000	Swant	X			X
CR	Rabbit	1:1000	Swant		X		
CR	Goat	1:1000	Swant				X
PV	Rabbit	1:1000	Swant			X	
PV	Guinea pig	1:500	Synaptic systems				X
vGAT	Mouse	1:500	Synaptic systems	X	X	X	X
GAD65	Guinea pig	1:500	Synaptic systems	X	X	X	
GAD67	Goat	1:100	R&D systems	X	X	X	

*Rows marked in the experiment column indicates the antigens labeled in each immunohistochemistry assay. See the “Materials and Methods” section for information on how the specificity of each antibody was verified*.

### *In Situ* Hybridization

*In situ* hybridization probes were designed by Advanced Cell Diagnostics, Inc. (Hayward, CA, USA) to detect mRNAs encoding GAD65 (*GAD2* gene), GAD67 (*GAD1* gene), CB (*CALB1* gene), CR (*CALB2* gene), or PV (*PVALB* gene). Tissue samples were processed using the RNAscope^®^ 2.0 Assay according to the manufacturer’s protocol. Briefly, tissue sections (12 μm) from the fresh-frozen right PFC of five subjects were fixed for 15 min in ice-cold 4% paraformaldehyde, incubated in a protease treatment, and then the probes were hybridized to their target mRNAs for 2 h at 40°C. The sections were exposed to a series of incubations that amplified the target probes, and then counterstained with DAPI. GAD65 and GAD67 mRNAs were detected with Alexa 488 and Atto 647, respectively. CB, CR, or PV mRNA was detected with Atto 550.

### Microscopy

Data from immunohistochemistry experiments were collected on an Olympus (Center Valley, PA, USA) IX81 inverted microscope equipped with an Olympus spinning disk confocal unit, Hamamatsu EM-CCD digital camera (Bridgewater, NJ, USA), and high precision BioPrecision2 XYZ motorized stage with linear XYZ encoders (Ludl Electronic Products Ltd., Hawthorne, NJ, USA) using a 60× 1.40 N.A. SC oil immersion objective. The equipment was controlled by SlideBook 6.0 (Intelligent Imaging Innovations, Inc., Denver, CO, USA), which was the same software used for post-image processing. 3D image stacks (2D images successively captured at intervals separated by 0.25 μm in the z-dimension) that were 512 × 512 pixels (~137 × 137 μm; pixel size = 0.267 μm) were acquired over 50 percent of the total thickness of the tissue section starting at the coverslip. Importantly, imaging the same percentage, rather than the same number of microns, of the tissue section thickness controls for the potential confound of storage and/or mounting related volume differences (i.e., *z*-axis shrinkage). The stacks were collected using optimal exposure settings (i.e., those that yielded the greatest dynamic range with no saturated pixels), with differences in exposures normalized during image processing.

### Sampling

As determined by measurements made in Nissl-stained sections, the boundaries of the six cortical layers were estimated based on the distance from the pial surface to the white matter: 1 (pia-10%), 2 (10%–20%), 3 (20%–50%), 4 (50%–60%), 5 (60%–80%) and 6 (80%-gray/white matter border). Ten systematic randomly sampled image stacks were taken in each layer per section by applying a sampling grid of 180 × 180 μm^2^.

### Image Processing

Each fluorescent channel was deconvolved using Autoquant’s Blind Deconvolution algorithm. Data segmentation was performed as previously described (Rocco et al., 2016a,b, 2017). Briefly, a Gaussian channel was made for each deconvolved channel by calculating a difference of Gaussians using sigma values of 0.7 and 2. The Gaussian channel was used for data segmentation only. The Ridler-Calvard iterative thresholding algorithm (Ridler and Calvard, 1978) was used to obtain an initial value for iterative segmentation for each channel within each image stack. Multiple iterations with subsequent threshold settings increasing by 25 gray levels were performed in MATLAB (R2015b). After each iteration, the object masks were size-gated within a range of 0.05–0.7 μm^3^. For analyses, the image stacks were virtually cropped in the x-, y- and z-dimensions using the center x-, y- and z-coordinates of the IR puncta object masks. In the x- and y-dimensions, the center of each object mask had to be contained in the central 490 × 490 pixels of the image. To select the z-dimension used for analyses, the z-position of each object mask was normalized by the following equation: Z_coordinate_ (# of z-planes for image stack/40).

Next, each object mask was placed in 1 of 40 z-bins based on its normalized z-position. The mean object mask density and mean fluorescence intensity for vGAT and GAD67 were determined within each z-bin, and used for an analysis of variance with *post hoc* comparison via Tukey’s honestly significant difference test. The maximum number of adjacent z-bins that were not significantly different for both intensity and object mask number across all channels were used for analyses. By taking this approach we controlled for possible edge effects (i.e., all puncta assessed were fully represented in the virtual space), differences in antibody penetration and differences in fluorochromes. The final object masks were then used to collect information on the deconvolved channels and to determine terminal density.

### Lipofuscin in Human Postmortem Brain Tissue

The major source of native fluorescence in postmortem tissue is from lipofuscin, an intracellular lysosomal protein that accumulates with age (Benavides et al., 2002; Porta, 2002) and fluoresces across the visible spectrum. In previous triple-label studies, we imaged lipofuscin in a fourth visible channel and during processing used information in the lipofuscin channel to exclude signal in the other channels for analysis. This approach has proven to be very effective (Sweet et al., 2010; Curley et al., 2011; Glausier et al., 2014; Rocco et al., 2016a, 2017). In the present studies, all visible channels were needed to separate four different proteins in the same section. Our spectral analysis of lipofuscin revealed that it has a broad Stokes shift such that upon being excited at 402 nm the emission signal can be efficiently collected at 705 nm. Thus, to eliminate this potential confound lipofuscin was imaged using a custom filter combination (402 ex/705 em) in a 5th channel. Lipofuscin was masked using an optimal threshold value, and mask objects made from the other channels that overlapped a lipofuscin mask were eliminated from analyses.

### Classification of Terminals

For immunohistochemistry experiments (Table 1), PV-IR, CB-IR and CR-IR puncta were classified as a terminal if they also contained vGAT and GAD65 and/or GAD67. A multistep process was used to classify vGAT-IR puncta as GAD65+, GAD67+, or GAD65/GAD67+ terminals. Specifically, mask operations were used to identify GAD65 and vGAT object masks that overlapped each other’s centers and did not overlap a GAD67 object mask (GAD65+ terminals). A similar approach was used to define GAD67+ terminals. vGAT object masks that overlapped the centers of both GAD65 and GAD67 object masks were defined as GAD65/GAD67+.

### Classification of Somatic GAD mRNA Content

The number of GAD65 and GAD67 mRNA molecules per GABAergic neuron was quantified. GABAergic neurons that contained ≥5 GAD65 mRNA or GAD67 mRNA molecules (>2.5X the density of GAD65 mRNA or GAD67 mRNA molecules in the neuropil) were considered to specifically express that transcript.

### Statistics

All analyses were performed on the mean values for individual subjects. The density and percentage of each GAD+ terminal subpopulation arising from the different GABAergic neuron subtypes were determined as follows: (1) the values of each measure were averaged for each image stack; (2) the stack means were averaged within layer; (3) layer means were averaged within section; and (4) the section averages were used to generate the mean (± standard deviation [SD]) density and percentage of each GAD+ terminal subpopulation per subject. The density of each GAD+ terminal subpopulation was assessed using analysis of variance with *post hoc* comparison via Tukey’s honestly significant difference test. For analyses with unequal variances, between groups* post hoc* comparison was performed via the Dunnett T3 test.

## Results

### The Proportion of vGAT+ Terminals Containing CB, CR, or PV Differs across Cortical Layers

**Figure 1:**
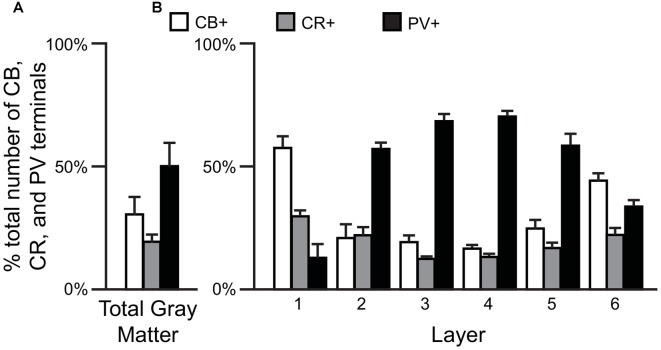
The proportion of GABAergic calbindin+ (CB+), calretinin+ (CR+) and parvalbumin+ (PV+) terminals differs across cortical layers. **(A,B)** Bar graphs showing the relative proportions of vesicular GABA transporter (vGAT) terminals in prefrontal cortex (PFC) that were CB+, CR+ or PV+. Error bars = SEM.

Comparisons of the relative proportions of vGAT terminals in total gray matter of the human PFC (*N* = 5) revealed that ~50% of the terminals were PV+, ~31% CB+ and ~19% CR+ (*F*_(2,12)_ = 74.6, *p* < 0.0005; Figure 1A). A laminar analysis found that these proportions differed for each of the terminal subtypes across the six cortical layers (CB *F*_(5,24)_ = 21.58, *p* < 0.0005; CR *F*_(5,24)_ = 7.96, *p* < 0.0005, and PV *F*_(5,24)_ = 38.42, *p* < 0.0005). The biggest differences from the findings in total gray matter were in layers 1 and 6 where CB+ terminals represented ~57% and ~44% of the terminals, respectively. In addition, CR+ terminals represented ~30% of the total in layer 1. In contrast, on average 64% of the terminals in layers 2–5 were PV+ (Figure 1B).

### Densities of Three Types of CB Terminals Differ across Layers

**Figure 2:**
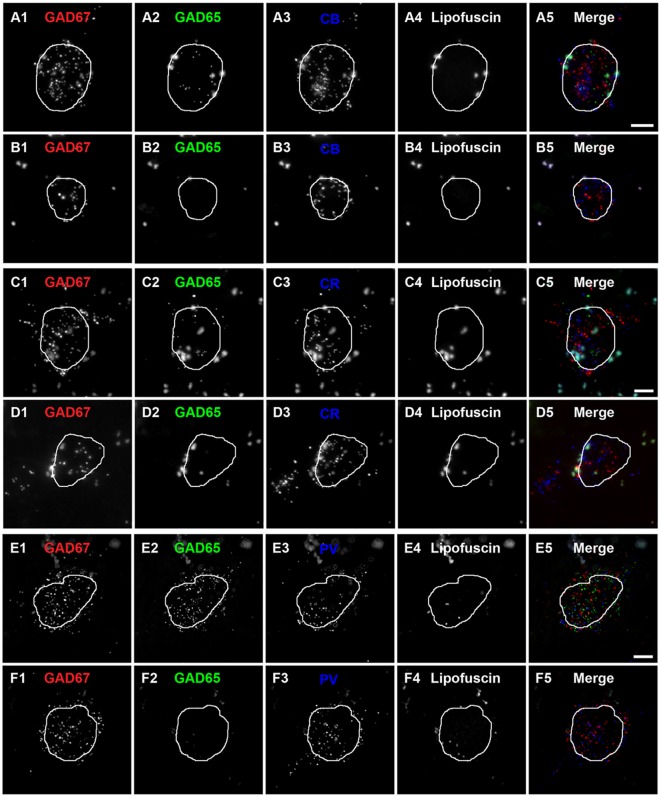
Subtypes of CB, CR and PV neurons are distinguishable by the expression of GAD65 mRNA in human PFC.**(A,B)** Single plane image of a PFC tissue section labeled for CB, GAD65 and GAD67 mRNAs. **(A5)** and **(B5)** are merged images of **(A1–A3)** and **(B1–B3)**, respectively. Lipofuscin autofluorescence is shown in **(A4,B4)**. **(C,D)** Single plane image of a PFC tissue section labeled for CR, GAD65 and GAD67 mRNAs. **(C5)** and **(D5)** are merged images of **(C1–C3)** and **(D1–D3)**, respectively. Lipofuscin autofluorescence is shown in **(C4,D4)**. **(E,F)** Single plane image of a PFC tissue section labeled for PV, GAD65 and GAD67 mRNAs.**(E5)** and **(F5)** are merged images of **(E1–E3)** and **(F1–F3)**, respectively. Lipofuscin autofluorescence is shown in **(E4,F4)**. In all images, the outline of the nucleus, which was visualized using DAPI, is shown. Scale bars = 5 μm.

**Figure 3:**
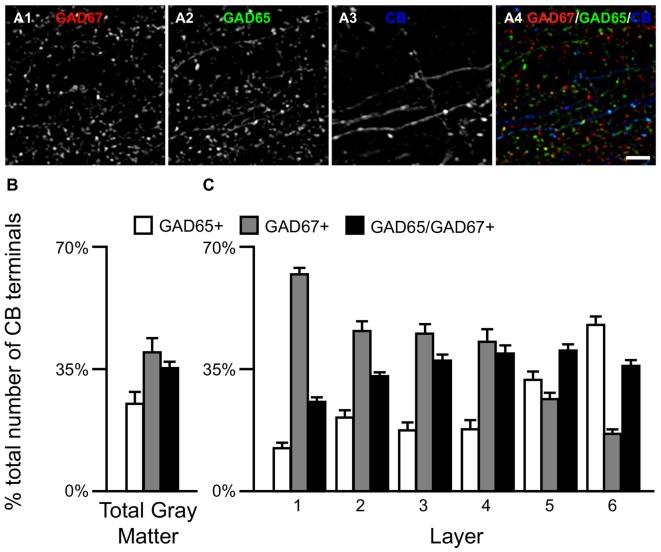
The proportion of three CB/GAD+ terminal subpopulations differs across cortical layers. **(A)** Projection image (6 z-planes separated by 0.25 μm) of a human PFC tissue section immunolabeled for CB, vGAT (not shown), GAD65 and GAD67. Gray scale images **(A1–A3)** are single channel images of the multichannel image **(A4)** and are representative of the immunohistochemistry labeling. Scale bar = 5 μm. **(B,C)** Bar graphs showing the relative proportions of vGAT/CB+ terminals in PFC that were GAD65+, GAD67+, or GAD65/GAD67+. Error bars = SEM.

Qualitative assessment revealed that terminals containing vGAT and CB contained either GAD65 or GAD67 or both (Rocco et al., 2016b). At the cell level, GABA neurons that expressed CB mRNA contained either both GAD65 and GAD67 mRNAs (Figures 2A1–A5) or only GAD67 mRNA (Figures 2B1–B5). Quantitative analysis (*N* = 20) found that the percentage of CB/GAD+ terminals that were GAD65+, GAD67+ or GAD65/GAD67+ differed (*F*_(2,57)_ = 15.1, *p* < 0.0005) (Figure 3). Specifically, ~40% of CB GABA terminals contained GAD67, ~35% contained both GAD proteins, and ~25% contained GAD65 in total gray matter. However, a laminar analysis found that these proportions differed for each of the terminal subtypes across the six cortical layers (CB/GAD65+ *F*_(5,114)_ = 48.10, *p* < 0.0005; CB/GAD67+ *F*_(5,114)_ = 39.13, *p* < 0.0005; and CB/GAD65/GAD67+ *F*_(5,114)_ = 14.06, *p* < 0.0005). The biggest differences from the findings in total gray matter were in layers 1 and 6 where CB/GAD67+ terminals represented ~62% and CB/GAD65+ terminals represented ~48% of the terminals, respectively. In addition, in layers 5 and 6 the CB/GAD67+ terminals constituted only 26% and 16%, respectively, of all CB+ GABA terminals.

We next assessed the relative levels of each GAD protein, as reflected in fluorescence intensity, in CB+ axon terminals. The relative amount of GAD65 in CB/GAD65+ terminals (6887 ± 1726 arbitrary units (a.u.)) was ~34% greater (*t*_(33)_ = 3.8, *p* = 0.001) than in CB/GAD65/GAD67+ terminals (5129 ± 1146 a.u.), whereas the relative amount of GAD67 was ~24% greater (*t*_(38)_ = 3.3, *p* = 0.002) in CB/GAD65/GAD67+ terminals (5569 ± 1069 a.u.) than in CB/GAD67+ terminals (4479 ± 1036 a.u.). These relative intensity values might not correspond directly to total protein in a terminal given that larger terminals will generally have more protein content and therefore, a greater amount of total fluorescence intensity. In fact, the volumes of the different subpopulations of terminals were significantly different (*F*_(2,57)_ = 287, *p* < 0.0005), with mean terminal volume greatest for CB/GAD65/GAD67+ terminals (0.52 ± 0.019 μm^3^), intermediate for CB/GAD67+ terminals (0.41 ± 0.033 μm^3^) and smallest for CB/GAD65+ terminals (0.32 ± 0.024 μm^3^). As is indicative from the above, CB+ terminals containing both GADs contained 32% more total GAD65 than CB/GAD65+ terminals and 60% more total GAD67 than CB/GAD67+ terminals.

Finally, we quantified terminal vGAT levels. The total amount of vGAT protein in the different subpopulations of CB/GAD+ terminals differed significantly (*F*_(2,57)_ = 79.1, *p* < 0.0005). *Post hoc* analysis showed that the total amount of vGAT in CB/GAD65/GAD67+ terminals was 39% greater (*p* < 0.0005) than in CB/GAD67+ terminals, which contained 48% more (*p* < 0.0005) vGAT protein than CB/GAD65+ terminals.

### Densities of Three Types of CR Terminals Differ across Layers

**Figure 4:**
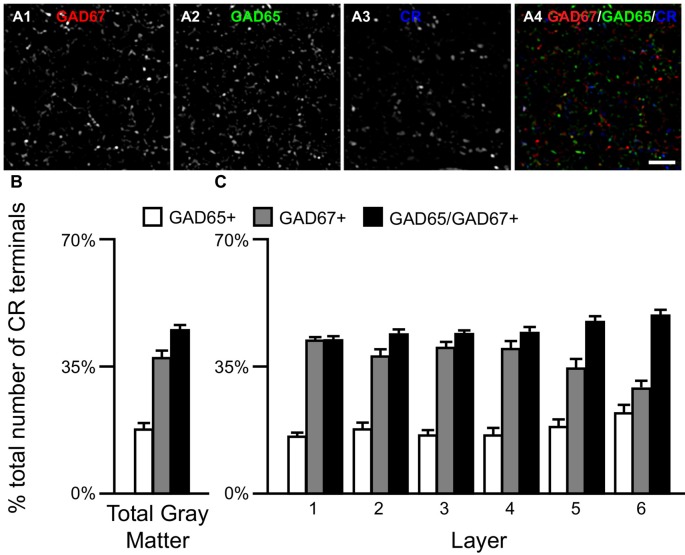
The proportion of three CR/GAD+ terminal subpopulations differs across cortical layers. **(A)** Projection image (7 z-planes separated by 0.25 μm) of a human PFC tissue section immunolabeled for CR, vGAT (not shown), GAD65 and GAD67. Gray scale images **(A1–A3)** are single channel images of the multichannel image **(A4)** and are representative of the immunohistochemistry labeling. Scale bar = 5 μm. **(B,C)** Bar graphs showing the relative proportions of vGAT/CR+ terminals in PFC that were GAD65+, GAD67+, or GAD65/GAD67+. Error bars = SEM.

Similar to CB neurons, CR neurons give rise to three distinct terminal subpopulations: CR/GAD65+, CR/GAD67+ and CR/GAD65/GAD67+ terminals (Rocco et al., 2016b). In addition, GABA neurons that expressed CR mRNA expressed both GAD65 and GAD67 mRNAs (Figures 2C1–C5) or only GAD67 mRNA (Figures 2D1–D5). Quantitative analysis (*N* = 20) found that the percentage of CR/GAD+ terminals represented by each subpopulation differed (*F*_(2,57)_ = 34.97, *p* < 0.0005) in total gray matter such that ~18% of CR GABA terminals contained GAD65, ~37% contained GAD67, and ~45% contained both GAD proteins (Figure 4). A laminar analysis found no difference in the percentage of CR+ terminals that were GAD65+ between the different cortical layers. In contrast, the percentage of CR+ terminals that contained GAD67 or both GAD proteins differed across the six cortical layers (CR/GAD67+ *F*_(5,114)_ = 8.63, *p* < 0.0005; CR/GAD65/GAD67+ *F*_(5,114)_ = 4.88, *p* < 0.0005). These differences were largely due to layers 5–6 where the percentage of CR+ terminals containing GAD67 was lower than in the other layers and the percentage that contained both GAD proteins was higher.

We next assessed the levels of each GAD protein in CR+ axon terminals. The total amount of GAD65 in CR/GAD65/GAD67+ terminals (77366 ± 17181 a.u.) was ~41% greater (*t*_(38)_ = 4.7, *p* < 0.0005) than in CR/GAD65+ terminals (55043 ± 12751 a.u.), whereas the total amount of GAD67 was ~63% greater (*t*_(38)_ = 7.2, *p* < 0.0005) in CR/GAD65/GAD67+ terminals (58025 ± 11443 a.u.) than in CR/GAD67+ terminals (35638 ± 7783 a.u.). As suggested by these findings, the total amount of vGAT protein in the different subpopulations of CR/GAD+ terminals differed significantly (*F*_(2,57)_ = 50.1, *p* < 0.0005). Specifically, the total amount of vGAT in CR/GAD65/GAD67+ terminals was 53% greater (*p* < 0.0005) than in CR/GAD67+ terminals, which contained 12% more (*p* = 0.256) vGAT protein than CR/GAD65+ terminals.

### Densities of Two Types of PV Terminals Differ across Layers

**Figure 5:**
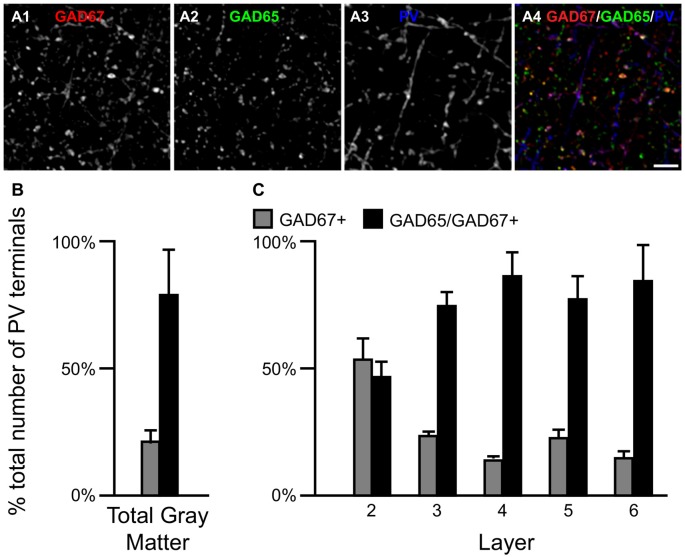
The proportion of two PV/GAD+ terminal subpopulations differs across cortical layers. **(A)** Projection image (5 z-planes separated by 0.25 μm) of a human PFC tissue section immunolabeled for PV, vGAT (not shown), GAD65 and GAD67. Gray scale images **(A1–A3)** are single channel images of the multichannel image **(A4)** and are representative of the immunohistochemistry labeling. Scale bar = 5 μm. **(B,C)** Bar graphs showing the relative proportions of vGAT/PV+ terminals in PFC that were GAD67+ or GAD65/GAD67+. Error bars = SEM.

Most PV neurons are chandelier or basket cells, which can be differentiated by the cells and perisomatic region they target. A qualitative assessment of GAD protein in PV+ terminals identified two distinct subpopulations: (1) PV/GAD67+; and (2) PV/GAD65/GAD67+ (Fish et al., 2011; Glausier et al., 2014). At the cell level, PV mRNA containing neurons expressed either mRNA encoding both GAD65 and GAD67 or only GAD67 mRNA (Figures 2E1–E5, 2E1–E5, F1–F5, respectively). The percentage of PV+ terminals that contained both GAD proteins (~78%) differed (*t*_(8)_ = 5.4, *p* = 0.001) from the percentage that contained only GAD67 protein (~22%; Figure 5) in total gray matter (*N* = 5). A laminar analysis, which only assessed layers 2–6 because only a small percentage (~5%) of all PV+ terminals were in layer 1, found that this difference was present in layers 3–6 but not in layer 2.

We next assessed the level of GAD67 protein in PV+ axon terminals. The total amount of GAD67 in PV/GAD65/GAD67+ terminals (30277 ± 2693 a.u.) was ~99% greater (*t*_(8)_ = 7.7, *p* < 0.0005) than in PV/GAD67+ terminals (15197 ± 3438 a.u.). As suggested by these findings, the total amount of vGAT protein in PV/GAD65/GAD67+ terminals was 157% greater (*t*_(8)_ = 18.9, *p* < 0.0005) than in PV/GAD67+ terminals.

## Discussion

The goal of the present study was to use multi-label immunohistochemistry and quantitative fluorescence confocal microscopy to gain insight into the types and amount of inhibition each cortical layer in the human PFC is likely to receive. Our findings suggest that each cortical layer receives a unique type (based on GAD proteins) and amount (based on the number of inhibitory synapses) of inhibition from GABAergic CB, CR and PV neurons that may reflect the intra- and inter-laminar processing demands required for proper PFC functioning.

Similar to our previous findings in the macaque monkey PFC (Fish et al., 2011, 2013; Rocco et al., 2016b), we found in the human PFC three subpopulations of vGAT terminals based on GAD content: GAD65+, GAD67+ and GAD65/GAD67+. Analyses of CB+, CR+ and PV+ terminals revealed that both CB+ and CR+ terminals comprised all three GAD subsets (GAD65+, GAD67+, or GAD65/GAD67+), whereas PV+ terminals comprised only two subsets (GAD67+ or GAD65/GAD67+). Multiplex mRNA labeling revealed that based on GAD mRNA expression CB, CR, and PV GABA neurons can be divided into two subpopulations: those containing only GAD67 mRNA and those containing both GAD65 and GAD67 mRNAs. Thus, it would appear in human PFC that GAD67+ terminals arise from neurons that express only GAD67 mRNA, whereas GAD65+ and GAD65/GAD67+ terminals arise from neurons expressing both GAD65 and GAD67 mRNAs. Several possibilities might explain the latter finding. First, it is possible that the postsynaptic target influences terminal GAD content. For example, terminals from PV basket cells, which target the soma and proximal dendrites of PCs, contain both GADs. In contrast, terminals from PV chandelier cells, which exclusively target the axon initial segment of PCs, contain only GAD67 (Fish et al., 2011, 2013; Glausier et al., 2014). The latter finding may be a primate specific event due to hypermethylation of the GAD2 gene (Luo et al., 2017). The lack of GAD67 in some terminals might reflect differences in GAD65 and GAD67 trafficking and half-life. For example, because GAD65 has a very long half-life (>24 h) and is efficiently trafficked to axonal terminals, every terminal from a neuron expressing mRNA for both GADs would be expected to have detectable levels of GAD65. In contrast, the short half-life (~2 h) of GAD67, along with a trafficking mechanism that is partially dependent on GAD65 (Kanaani et al., 2010), might result in undetectable levels of GAD67 in some terminals. In addition, changes in network activity alter GAD67 protein levels (i.e., decreased activity leads to a decrease in GAD67 protein; Lau and Murthy, 2012). Thus, PC-GABA neuron subnetwork activity might differentially affect GAD67 terminal protein levels within a particular GABA neuron subtype. In support of this latter idea, in all of the three cell types assessed, we found that terminals containing only GAD65 had significantly lower amounts of vGAT protein, whose expression like GAD67 is activity-dependent, relative to those containing both GAD65 and GAD67 protein.

We previously showed that in layer 4 of the monkey PFC terminals arising from cannabinoid receptor 1 expressing basket (CB1rB) neurons contained GAD65, but had undetectable levels of GAD67 and GABA transporter 1 (GAT1). We proposed that lower GAT1 levels in CB1rB neuron terminals allowed for GABA release from them to act on receptors located outside the synaptic cleft. In support of this idea, CB1rB neuron vesicular GABA release can spillover to affect PV synapses in close proximity (Karson et al., 2009) as well as extra-synaptic GABA_A_ receptors (Alle and Geiger, 2007; Karson et al., 2009). Future studies that assess GAT1 levels in CB+ and CR+ terminals that contain GAD65, but not GAD67, are needed to determine if there is a relationship between GAD and GAT1 terminal expression levels.

**Figure 6:**
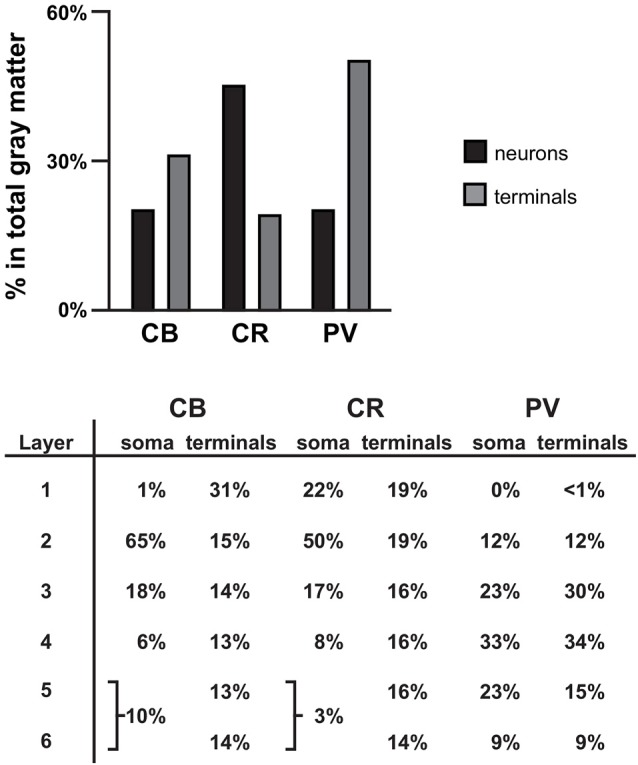
Summary. Bar graph showing the percentage of all γ-aminobutyric acid (GABA) neurons (black bars) and terminals assessed (gray bars) that is CB+, CR+, or PV+ in PFC total gray matter. Table showing the distribution of soma and terminals for each of the three GABA neuron subtypes across the six cortical layers. The soma data for CB and CR were taken from previously published findings in human PFC (Daviss and Lewis, 1995).

CB, CR and PV neurons play different roles in the local cortical network. Thus, the amount and type of GABA inhibition each cortical layer receives directly affects the integration of inputs to that layer (i.e., inhibition provided by CB and CR neurons) or the functional output of neurons located within the layer (i.e., inhibition provided by PV neurons). In primate PFC, CB, CR and PV neurons have distinct laminar distribution patterns (see Figures 1, 6 and Condé et al., 1994; Hof et al., 1999). The majority (~65% see Figure 6) of CB neurons, which makeup ~20% of all GABA neurons in primate PFC, are located in cortical layer 2 (Condé et al., 1994; Daviss and Lewis, 1995; Hof et al., 1999). In total gray matter we found that ~31% of the vGAT terminals assessed contained CB and ~31% of all CB+ terminals were located in layer 1. This finding suggests that many of the CB+ terminals arise from distal dendrite-targeting Martinotti cells, which are defined by their ascending axonal projections that span multiple layers and ramify in layer 1 (Fairén et al., 1984). Somata of CB-expressing Martinotti cells are found across layers 2–5 (Wang et al., 2004). Martinotti cells have been shown to provide feedback inhibition between neighboring PCs in layer 5 (Silberberg and Markram, 2007), the main cortical output layer, which may serve to synchronize the activity of subtypes of subcortical projecting neurons (Hilscher et al., 2017). Another subtype of CB neuron present in primates, the horse-tail cell, has its somata located in layers 2 through superficial 3. These cells give rise to tightly-bundled descending axonal projections that terminate within a narrow column across multiple deeper layers. Horse-tail cells are distributed at regular intervals and are thought to contribute to the microcolumnar organization within the primate neocortex (Defelipe et al., 1990; del Río and DeFelipe, 1995; Peters and Sethares, 1997); though, their function has been difficult to study due to their apparent absence in other mammalian species (Yáñez et al., 2005). In human cortex >40% of CB cells express the neuropeptide somatostatin (SST) and >80% of SST neurons express CB (González-Albo et al., 2001). SST-expressing neurons are a morphological diverse subtype that includes both Martinotti and horse-tail cells (González-Albo et al., 2001; Wang et al., 2004). In addition to being expressed by neuronal subtypes that terminate across multiple layers, synaptic connections from other subtypes of SST neurons are relatively confined to the same layer where their soma is located (Ma et al., 2006), where they exert control over layer-specific microcircuits, such as those involved in processing thalamic inputs to layer 4 (Xu et al., 2013). Moreover, it was recently demonstrated that their laminar location influences their function (Muñoz et al., 2017).

Although in primate PFC ~45% of GABAergic neurons contain CR, only 19% of the vGAT terminals assessed contained CR, similar to our finding in monkey PFC (Rocco et al., 2016b). The greatest density of CR neurons is in PFC layers 2-superficial 3 (Condé et al., 1994; Hof et al., 1999); however, we found that 16%–19% of all CR+ terminals were located in layers 1–5 and 14% were found in layer 6. Considering that CR GABAergic neurons mainly target dendrites of other GABAergic neurons (Melchitzky and Lewis, 2008), which are sparsely located across cortical layers, it is not surprising that CR+ terminals are relatively evenly distributed across cortical layers, and represent the lowest percentage of all GABAergic terminals relative to CB+ and PV+ terminals. In the PFC of monkey, >85% of CR neurons express the neuropeptide vasoactive intestinal polypeptide (VIP) and ~80% of VIP neurons express CR (Gabbott and Bacon, 1997). Within cortical microcircuits, VIP neurons mainly inhibit SST, and to a lesser extent, PV neurons (Pfeffer et al., 2013; Pi et al., 2013), suggesting that they play a strong role in many of the same microcircuits (Muñoz et al., 2017). By mainly synapsing onto other GABAergic neurons, CR neurons mediate disinhibitory control of PCs, which leads to selective amplification of local signal processing (Pi et al., 2013).

In primate PFC ~20% of GABAergic neurons express PV. However, in total gray matter we found that ~50% of terminals expressing a calcium binding protein contained PV. This finding is supported by evidence that on average PV neurons make more contacts with PCs than other GABA neurons and also heavily innervate each other (Markram et al., 2004; Pfeffer et al., 2013). PV neurons provide perisomatic input to PCs in the same layer and to lesser extent to PCs located in layers directly above and below. Considering that approximately half of PV neurons are located in PFC layers 3–4, it is not surprising that we found ~64% of all PV+ terminals in these layers. The high density of PV innervation within middle cortical layers is important for generating gamma oscillations (Klausberger and Somogyi, 2008; Cardin et al., 2009) and processing feedforward afferent inputs (Xu et al., 2013). Finally, ~20% of PV+ terminals only contained GAD67 protein (presumed chandelier cell terminals), with the greatest density of these terminals in layer 2. This percentage/distribution is consistent with other studies of chandelier cells (Defelipe et al., 1989).

Feedforward cortico-cortical and thalamo-cortical projections target distinct layers within the PFC (Giguere and Goldman-Rakic, 1988; Barbas and Rempel-Clower, 1997). The innervation pattern of CB+, CR+ and PV+ terminals is apparently well-equipped for regulating these layer-specific inputs, as well as for generating and maintaining synchrony within local networks via feedback inhibition in order to maintain proper PFC functioning. Finally, our findings suggest that the layer-specific distribution of CB+, CR+ and PV+ terminals are likely to differ across cortical regions with distinct laminar cytoarchitectures (e.g., granular vs. agranular regions) in a manner that would provide the needed complement of GABA inputs for the specific functional (e.g., primary sensory, association or motor) properties of each region.

## Author Contributions

KNF, BRR and DAL contributed intellectually to experimental design, data interpretation and writing of the manuscript. BRR and KNF performed the experiments.

## Conflict of Interest Statement

DAL currently receives investigator-initiated research support from Pfizer and has recently served as a consultant in the areas of target identification and validation and new compound development for Merck. The other authors declare that the research was conducted in the absence of any commercial or financial relationships that could be construed as a potential conflict of interest.
