# Supplementary material for: Laminar Distribution of Subsets of GABAergic Axon Terminals in Human Prefrontal Cortex
Source: Front Neuroanat. 2018 Feb 16;12:9. doi: 10.3389/fnana.2018.00009 (PMC5820353; doi:10.3389/fnana.2018.00009)
Supplement: Supplementary file 1 [file Table_1.pdf]

Supplemental Table 1

Demographic and postmortem characteristics of human subjects used in this study. *PMI=postmortem interval (hours); **Years stored in 30% glycerin/30% ethylene glycol solution at -30ºC.

**Case**	**Sex/Race**	**Age (Yrs)**	**PMI***	**Storage Time****	**Cause of Death**
727	M/B	19	7.00	12.90	Trauma
852	M/W	54	8.00	10.90	Cardiac tamponade
1307	M/B	32	4.80	5.20	ASCVD
567	F/W	46	15.00	14.80	Mitral valve prolapse
1047	M/W	43	13.80	8.10	ASCVD
739	M/W	40	15.8	13	ASCVD
451	M/W	48	12.00	16.30	ASCVD
178	M/W	48	7.80	20.50	ASCVD
452	F/W	40	14.30	16.30	ASCVD
449	F/W	47	4.30	16.30	Accidental CO poisoning
681	M/W	51	11.60	14.10	Hypertrophic cardiomyopathy
395	M/W	42	12.30	18.80	Pericardial tamponade
575	F/B	55	11.30	15.40	ASCVD
278	M/W	50	4.50	20.40	ASCVD
1284	M/W	55	6.40	5.60	ASCVD
1122	M/W	55	15.40	7.30	Cardiac Tamponade
250	F/W	47	5.30	19.70	ASCVD
412	M/W	42	14.20	17.50	Aortic stenosis
344	M/W	50	6.80	18.60	ASCVD
1391	F/W	51	7.8	4	ASCVD
**Mean (SD)**		45.75 (8.69)	9.92 (4.04)	13.78 (5.28)	
